# Picture quiz

**Published:** 2014

**Figure:**
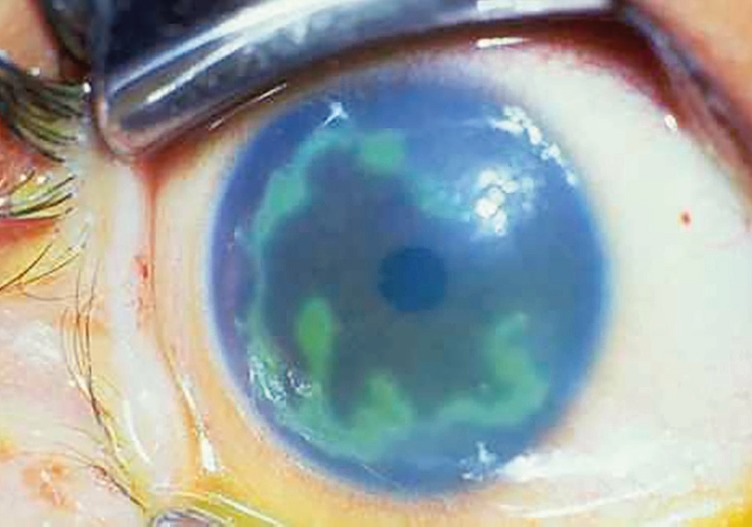


A 3 year-old child in Africa presented with a history of sore eyes following an illness with fever. There was no history of injury. Both eyes had similar findings.

1. What is the diagnosis? (Select one.)
  □ **a.** Ophthalmia neonatorum
  □ **b.** Fungal keratitis
  □ **c.** Episcleritis
  □ **d.** Herpes simplex ulcer
  □ **e.** Use of traditional eye medicines
2. Which of the fol lowing are known risk factors for the answer to question 1? (Select all that apply.)
  □ **a.** Measles
  □ **b.** HIV infection
  □ **c.** Malaria
  □ **d.** Iritis
  □ **e.** Malnutrition
3. Which of the following is the first line recommended treatment for the answer to question 1? (Select one.)
  □ **a.** Prednisolone drops
  □ **b.** Chloramphenicol ointment
  □ **c.** Acyclovir ointment
  □ **d.** Natamycin ointment
  □ **e.** Atropine drops

## ANSWERS

1. Diagnosis: d. Herpes simplex ulcer. This is a large dendritic/geographic ulcer caused by herpes simplex virus (HSV).
2. Risk factors: a, b, c, and e are possible. Measles and malaria can cause fever and suppress immunity, so both are risk factors for HSV infection. HIV infection and malnutrition can suppress immunity, so both of these are also risk factors for HSV infection.
3. Recommended treatment: c. Acyclovir ointment five times per day for 10 days is a suitable treatment for corneal ulceration of the epithelium caused by HSV.

**Note:** Steroids are contraindicated in dendritic/geographic ulcer.

